# Genetic and Morphological Divergence in Three Strains of Brook Trout *Salvelinus fontinalis* Commonly Stocked in Lake Superior

**DOI:** 10.1371/journal.pone.0113809

**Published:** 2014-12-05

**Authors:** Garrett J. McKinney, Anna Varian, Julie Scardina, Krista M. Nichols

**Affiliations:** 1 Purdue University, Department of Biological Sciences, West Lafayette, Indiana, United States of America; 2 Purdue University, Department of Forestry and Natural Resources, West Lafayette, Indiana, United States of America; University of Texas, United States of America

## Abstract

Fitness related traits often show spatial variation across populations of widely distributed species. Comparisons of genetic variation among populations in putatively neutral DNA markers and in phenotypic traits susceptible to selection (Q_ST_ F_ST_ analysis) can be used to determine to what degree differentiation among populations can be attributed to selection or genetic drift. Traditionally, Q_ST_ F_ST_ analyses require a large number of populations to achieve sufficient statistical power; however, new methods have been developed that allow Q_ST_ F_ST_ comparisons to be conducted on as few as two populations if their pedigrees are informative. This study compared genetic and morphological divergence in three strains of brook trout *Salvelinus fontinalis* that were historically or currently used for stocking in the Lake Superior Basin. Herein we examined if morphological divergence among populations showed temporal variation, and if divergence could be attributed to selection or was indistinguishable from genetic drift. Multivariate Q_ST_ F_ST_ analysis showed evidence for divergent selection between populations. Univariate analyses suggests that the pattern observed in the multivariate analyses was largely driven by divergent selection for length and weight, and moreover by divergence between the Assinica strain and each of the Iron River and Siskiwit strains rather than divergent selection between each population pair. While it could not be determined if divergence was due to natural selection or inadvertent artificial selection in hatcheries, selected differences were consistent with patterns of domestication commonly found in salmonids.

## Introduction

Fitness related traits often vary across populations of widely distributed species. Across its range, a species may be exposed to variation in both biotic and abiotic factors such as differences in resource availability, habitat structure, and temperature regimes that select for local adaptation, as well as repeatable temporal variation that may be unique to each population. As a result, selection may vary temporally during ontogeny as well as spatially across populations. Besides opportunities for divergent selection, spatial (or temporal) separation between populations also fosters reproductive isolation which allows for genetic drift to be a driver of divergence.

Evaluating whether quantitative trait differences are a result of selection is important in understanding whether adaptive differences exist between populations [1], [2]. Traditional methods for attributing morphological variation to drift or selection compare variation in neutral markers between populations (F_ST_) to the analogous measure of quantitative genetic variation underlying phenotypes between populations (Q_ST_) [3]. Since F_ST_ is based on neutral markers, F_ST_ can be viewed as the amount of neutral genetic divergence between populations and is used as a null expectation for the level of quantitative trait variation (Q_ST_) that is expected in the absence of selection [1]. Selection is inferred to be the cause of divergence when Q_ST_ estimates differ significantly from F_ST_ estimates.

Although comparisons of Q_ST_ to F_ST_ are an effective way to assess the cause of population differentiation, there are several limitations to traditional analyses. Q_ST_ F_ST_ comparisons typically require data from at least 10 populations to have the statistical power to differentiate drift and selection [4]. However, in many cases researchers do not have access to the number of populations necessary to achieve high statistical power or are more simply interested in whether local adaptation is occurring in a smaller number of specific populations. In addition, Q_ST_ F_ST_ comparisons cannot differentiate selection from drift when Q_ST_ and F_ST_ values are equal, even though selection can be acting [5]. Recently, a new Bayesian method, using the R package ‘driftsel’ has been developed for testing for neutrality of quantitative traits [5], [6]. Driftsel constructs a population-level coancestry matrix based on neutral marker data. From this matrix, a statistical distribution of the mean additive genetic values for an ancestral population is estimated. The distribution of the mean additive genetic values for the ancestral population is then used as a neutral expectation (analogous to F_ST_) against which the probability distribution of observed mean additive genetic values (analogous to Q_ST_) for each population can be compared to test if the observed pattern in quantitative trait differences could be generated by drift alone. As a result, driftsel has greater power for distinguishing between drift and selection, enabling comparisons between few populations as well as the ability to distinguish between drift and selection even in cases where Q_ST_ and F_ST_ are equal [5].

Brook trout in the Lake Superior basin have historically exhibited diverse ecological life history diversity, ranging from stream dwelling populations, to those that are born in streams and migrate to Lake Superior where they obtain very large sizes before returning to streams to spawn. The varied environments inhabited by *S. fontinalis* coupled with population subdivision provides an ideal situation for evolutionary change through natural selection in wild populations [7] but also creates ample opportunity for genetic drift to act on phenotypes [8]. In general, F_ST_ estimates in *S. fontinalis* populations show a moderate to high degree of population subdivision even for spatially close populations [9], [10].

Lake Superior was formerly home to robust populations of migratory book trout, however their numbers have greatly declined with only a handful of migrant populations remaining [11], [12]. Hatchery supplementation has in the past been used as a means to augment or attempt to restore populations, and is likely to continue to be a component of restoration efforts in the future [11], [13]. However, this practice often fails in creating self-sustaining populations or results in populations with reduced reproductive success, both with brook trout and with other salmonids [14]–[16]. This is often ascribed to use of hatchery stocks that are not locally adapted to the habitats where they are introduced. Numerous studies have examined fitness related traits and local adaptation in salmonids (for examples see [17]–[21]), however, few studies have utilized Q_ST_ F_ST_ analysis to determine whether quantitative trait diversity is a product of selection (but see [18], [22]).

In this study, multivariate quantitative trait divergence was examined in hatchery strains of brook trout (*Salvelinus fontinalis*) that were historically or currently used for stocking in the Lake Superior Basin, and vary in morphology and life history with both migratory and resident forms. We examined these populations to determine if extant diversity in morphology and size over several time points during development show patterns of variability consistent with neutral markers that are subject only to genetic drift, or if morphological traits show patterns of variability that are more consistent with patterns of natural or artificial selection.

## Materials and Methods

### Ethics statement

Work with the fish in this study was approved by the Purdue Animal Use and Care Committee (protocol ID 06-051).

### Fish strains and crosses

Three strains of brook trout (*S. fontinalis*) that were previously used to quantify heritability of morphological traits [23], and historically or currently used for stocking in the Lake Superior basin, were used for this study. These strains included the Siskiwit River, Assinica, and Iron River strains described below, and are representative of just some of the hatchery strains used for stocking in Lake Superior and surrounding streams. Siskiwit River brook trout are a migratory strain from the Big and Little Siskiwit Rivers on Isle Royale, Michigan [24]. The Siskiwit hatchery brood stock was founded with eight males and 11 females collected in 1995 and 1999, and was again supplemented with additional broodstock in 2004 [23]. The Siskwit strain was stocked for a short period of time in a number of Lake Superior tributaries in an attempt to revive the largely extirpated, migratory life history of brook trout (often called coaster brook trout), but currently it is rarely stocked in the basin. The Assinica strain was founded with four females and three males in 1962 in Lake Assinica, Quebec. These *S. fontinalis* were collected in late summer near the outlet of Lake Assinica and were presumed to be migrating from Lake Assinica into the Broadback River to spawn [25], [26]. Iron River *S. fontinalis* are a resident strain founded from 1,400 fish collected in 1993 from the Iron River, Michigan [27]. The Assinica and Iron River strains are currently used by the Michigan DNR for stocking in Michigan waters.

Crosses were created and used for a previous study on the heritability of morphological traits, and full details of mating design and rearing can be found therein [23]. In brief, parental fish were crossed using a combination of modified full-sib nested half-sib and partial factorial mating designs to allow partitioning of additive genetic variance for both heritability [23] and the Q_ST_ analyses herein. In general, each sire was used to fertilize two the three dams; in cases where egg lots from individual dams were large, eggs from single dams were split and fertilized by more than a single sire. Assinica and Iron River gametes were provided by the Michigan Department of Natural Resources, Marquette State Fish Hatchery (Marquette, Michigan). Gametes from the Siskiwit strain were provided by the U.S. Fish and Wildlife Service Iron River National Fish Hatchery (Iron River, Wisconsin). For the Assinica strain 30 families were created using 10 sires and 15 dams in a partial factorial breeding design. Twenty eight Iron River strain families were created from 10 sires and 20 dams in a partial factorial breeding design. Twelve Siskiwit strain families were produced from five sires and 12 dams in a modified full sib nested half sib design. Families were created on 09 and 15 November 2006.

### Neutral marker variation

To assess neutral marker variation, 1714 individuals from the three study populations were genotyped with fourteen microsatellite markers [28]; see Table S1. Samples sizes included 630 from Assinica, 353 from Siskiwit, and 731 from Iron River. All individuals used in the quantitative trait analyses were genotyped and used to assess neutral marker variation; however, not all genotyped individuals were used in quantitative trait analyses. DNA was extracted from fin clips (stored in 95% ethanol) using Wizard SV-96 genomic DNA kits (Promega, Madison, WI). Microsatellite loci were amplified and genotyped under the following conditions: initial denaturing of 94° for 2 min followed by 30 cycles of 94° for 45 sec, annealing temperature for 45 sec, and 72°C for 2 min with a final extension step at 72°C for 10 min. Annealing temperatures for individual loci are provided in Table S1. PCR products were combined with Liz500 size standard (Applied Biosystems Inc., Foster City, CA) and separated by size using electrophoresis on the ABI 3130xl (Applied Biosystems Inc., Foster City, CA). The genotypes, or size of the resulting amplification peaks were then visually scored for each individual using Genemapper v. 4.0 (Applied Biosystems Inc., Foster City, CA). Raw data for genotypes is available in Table S2.

### Quantitative traits

Sampling took place on three separate occasions: from 13–17 August 2007 (sampling period one), 22–26 October 2007 (sampling period two), and 21–24 January 2008 (sampling period three) [23]. At each time point, traits were measured on a subset of the total fish available. During sampling period one, 660 (40% of all fish alive at sampling period one) fish were sampled: 132 Siskiwit from 12 families, 256 Assinica from 27 families, and 257 Iron River from 20 families. During sampling period two, 496 (41% of the total) fish were sampled: 66 Siskiwit from 12 families, 232 Assinica from 27 families, and 198 Iron River from 20 families. During sampling period three, 348 (30% of the total) fish were sampled: 36 Siskiwit from 11 families, 183 Assinica from 26 families, and 129 Iron River from 20 families. Individuals were anesthetized with tricaine methanesulphonate (MS-222, Argent Chemicals, Redmond, WA), and fork length (mm) and wet weight (g) measurements were taken. Reflectance and body shape, described below, were also measured.

#### Reflectance

Reflectance is associated with smoltification in migrating salmonids where the pelagic habitat makes it advantageous to have a silvery coloration. Increased reflectance has been shown in anadromous *S. fontinalis* [29] and there have been differences in coloration detected in lacustrine *S. fontinalis* associated with habitat specialization [30]. It is currently unknown how the study populations herein may differ in reflectance, as two were derived from migratory or lake dwelling populations (Assinica and Siskiwit), while the third is entirely riverine (Iron River). Anesthetized fish were photographed by placing in a clear V-profile aquarium filled with water, as described by [31] and [23]. The aquarium was placed inside an enclosed, illuminated box to standardize picture conditions. The left side of the fish was photographed along with a metric scale to allow measurements to be taken. For the first and second time point a white and black patch were present on the tank to allow further standardization of light intensity, the third time point used a blue and white patch [31]. Camera settings were standardized for all photographs.

**Figure 1:**
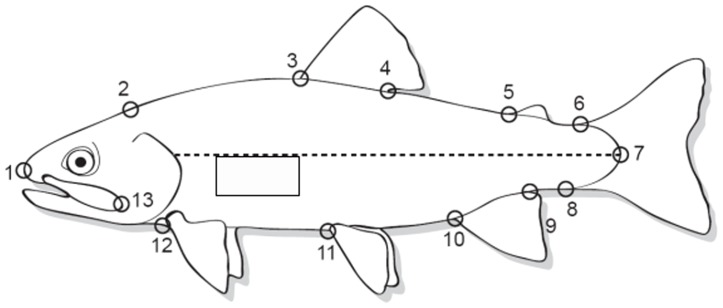
Location of region analyzed for skin reflectance (box) and placement of landmarks for analyzing body morphology (circles). Image modified with permission from [31] and [23].

Reflectance was quantified using SigmaScan Pro (San Jose, CA). The pixel intensity of the measured area was standardized by calibrating each picture with either black and white or blue and white patches on the acrylic box [31]. A rectangle was drawn at a consistent location on each fish as shown in Fig. 1; the average pixel intensity of this rectangle was calculated and used as a measure of reflectance. The reflectance scores were used for analyses of quantitative trait divergence (described below).

#### Body morphology

Body shape, evaluated by thin plates spline analysis [32], was quantified by relative warps scores previously described for the same families in a prior study [23]. The relative warps analysis was conducted using tpsRelw [32], as previously described by [23]. The six relative warps examined herein were all significantly heritable and explained 74.51% of total variation in body shape [23]. Briefly, thirteen landmarks were sequentially placed on each photograph to obtain body morphology measurements. Digitized landmarks were located at: 1) tip of snout, 2) top of gill arch placed on dorsal body axis, 3) anterior dorsal fin, 4) posterior dorsal fin, 5) anterior adipose fin, 6) anterior caudal fin on dorsal surface, 7) end of peduncle/lateral line, 8) anterior caudal fin on ventral surface, 9) posterior anal fin, 10) anterior anal fin, 11) anterior pelvic fin, 12) front of pectoral fin placed on ventral body axis, and 13) end of jaw placed on ventral body axis (Fig. 1). These relative warp scores were used as quantitative traits for analyses of quantitative trait divergence, together with length and weight, and reflectance values (see below). Raw data for morphological traits is contained in Tables S3, S4, and S5.

### Statistical analyses

#### Neutral marker variation (F_ST_)

F_ST_ between populations was calculated with Fstat [33] using the theta method [34], [35]. This estimate of F_ST_ was used to provide basic information on population differentiation and was not used further in the driftsel Q_ST_ F_ST_ analysis.

#### Quantitative trait variation

Mean trait values for length, weight, and morphology were previously summarized in [23]. For reflectance, differences in population mean values were tested using SAS proc glm.

#### Quantitative trait divergence analyses

The R package driftsel [6] was used to test the hypothesis that quantitative trait divergence is consistent with divergent natural selection, relative to neutral divergence estimated from putatively neutral molecular markers. Neutral divergence was estimated with the R package RAFM [36] using 200,000 iterations with 10,000 burnin iterations and a thinning interval of 100. Input data consisted of a matrix of all genotypes for all individuals for which quantitative traits were measured. Morphological divergence and neutrality tests were conducted with the R package driftsel [5] using 30,000 iterations with 10,000 burnin iterations and a thinning interval of 50. The posterior distribution of the coancestry matrix θ^P^ generated by RAFM was used as the prior for the function MH() in driftsel [6]. Input data consisted of a matrix of all traits for all individuals as well as a pedigree matrix listing the parents and parental populations for each sample. Convergence was tested by running three parallel chains for each analysis. Gelman-Rubin diagnostics were performed using the coda package in R [37] and confirmed that the output for each chain was indistinguishable, had converged for both neutral markers and morphological data, and showed no autocorrelation. Analyses in driftsel were conducted separately for each time point, whereby data at each time point from all populations and all traits was combined for a single multivariate analysis. The driftsel test statistic, S, does not compare estimates of Q_ST_ to a distribution of F_ST_ as has been done in traditional Q_ST_-F_ST_ analyses [2], [38], but describes the joint posterior probability that the observed pattern of population divergence arose under genetic drift. When S∼1 the observed pattern of population divergence is unlikely under neutrality and indicates divergent selection. When 0<S<1 then a typical neutral pattern is indicated, and when S∼0 this also indicates departure from neutrality but for stabilizing selection. In prior simulations from the authors that developed driftsel [5], [6], an S value of 0.8 was used as the credibility level to indicate divergent selection and 0.2 to indicate stabilizing selection to optimize specificity and sensitivity. To be more conservative, an S value credibility value of 0.9 was used to indicate divergent selection and 0.1 was used to indicate stabilizing selection for this study. Because driftsel, when considering multiple traits and populations, does not give a clear picture on which traits or populations are driving divergence in quantitative traits, we also ran driftsel with the above parameters for each trait at each time point individually, with different population groupings. Finally, the viz.traits function in driftsel was used to evaluate the patterns of quantitative trait divergence in multivariate and univariate analyses.

## Results

### Neutral marker variation (F_ST_)

F_ST_ estimates for the population comparisons are as follows: global F_ST_ was 0.164, F_ST_ between Iron River and Assinica was 0.1453, F_ST_ between Siskiwit and Assinica was 0.2, and F_ST_ between Siskiwit and Iron River was 0.1627. These F_ST_ values indicate moderate divergence in neutral markers and are in the range previously found between other, natural populations of Lake Superior *S. fontinalis* [39]. Raw genotype data used for this and in driftsel analyses are provided in Table S2.

### Quantitative trait variation

**Figure 2:**
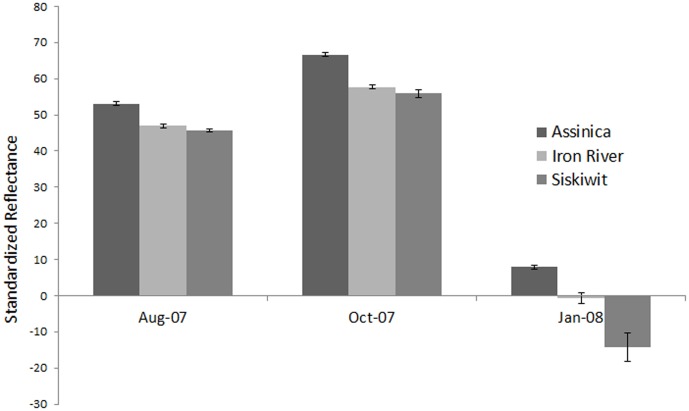
Standardized reflectance for each strain across sampling time points. *Indicates that all three strains are significantly different. **Indicates that Assinica is significantly different from both Siskiwit and Iron River. Magnitude of reflectance for the last time point is not directly comparable to the first two as a different intensity standard was used.

Length, weight, and morphological trait variation have been previously described in these samples [23]. The general trends observed for these traits were that the Assinica strain was larger in both length and weight at all time points, when compared to Iron River and Siskiwit [23]. This trend was also observed in morphology, where Assinica were consistently different in body shape at all time points. Assinica generally had deeper bodies and caudal peduncles, shorter heads, and mouths oriented in a more dorsal position than the Iron River and Siskiwit strains [23]. Reflectance, the additional trait added in this study in these populations, varied over time and by population. At the first time point reflectance was significantly different among strains (F_2,651_ = 67.89, p<0.001) (Fig. 2). The Assinica strain had greater reflectance, indicating more silvering which is associated with smoltification, and was significantly different from both Siskiwit (p<0.0001) and Iron River (p<0.001); Iron River and Siskiwit were not significantly different from each other (p = 0.259). Reflectance at the second time point was also different among strains (F_2,484_ = 105.25, p<0.001). The Assinica strain had greater reflectance and was significantly different from both Siskiwit (p = 0.001) and Iron River (p = 0.001), Iron River and Siskiwit were not significantly different from each other (p = 0.198). At the third time point, reflectance was significantly different among strains (F_2,329_ = 20.43, p<0.001), and was significantly different in all possible pairwise comparisons between populations (Assinica-Siskiwit p<0.001, Assinica-Iron River p<0.001, Iron River-Siskiwit p = 0.002). The Assinica strain had the greatest reflectance, followed by Iron River and Siskiwit. Reflectance increased between the first and second time point for all populations, and the third time point is not directly comparable as a different intensity standard was used.

### Quantitative trait divergence analyses

**Figure 3:**
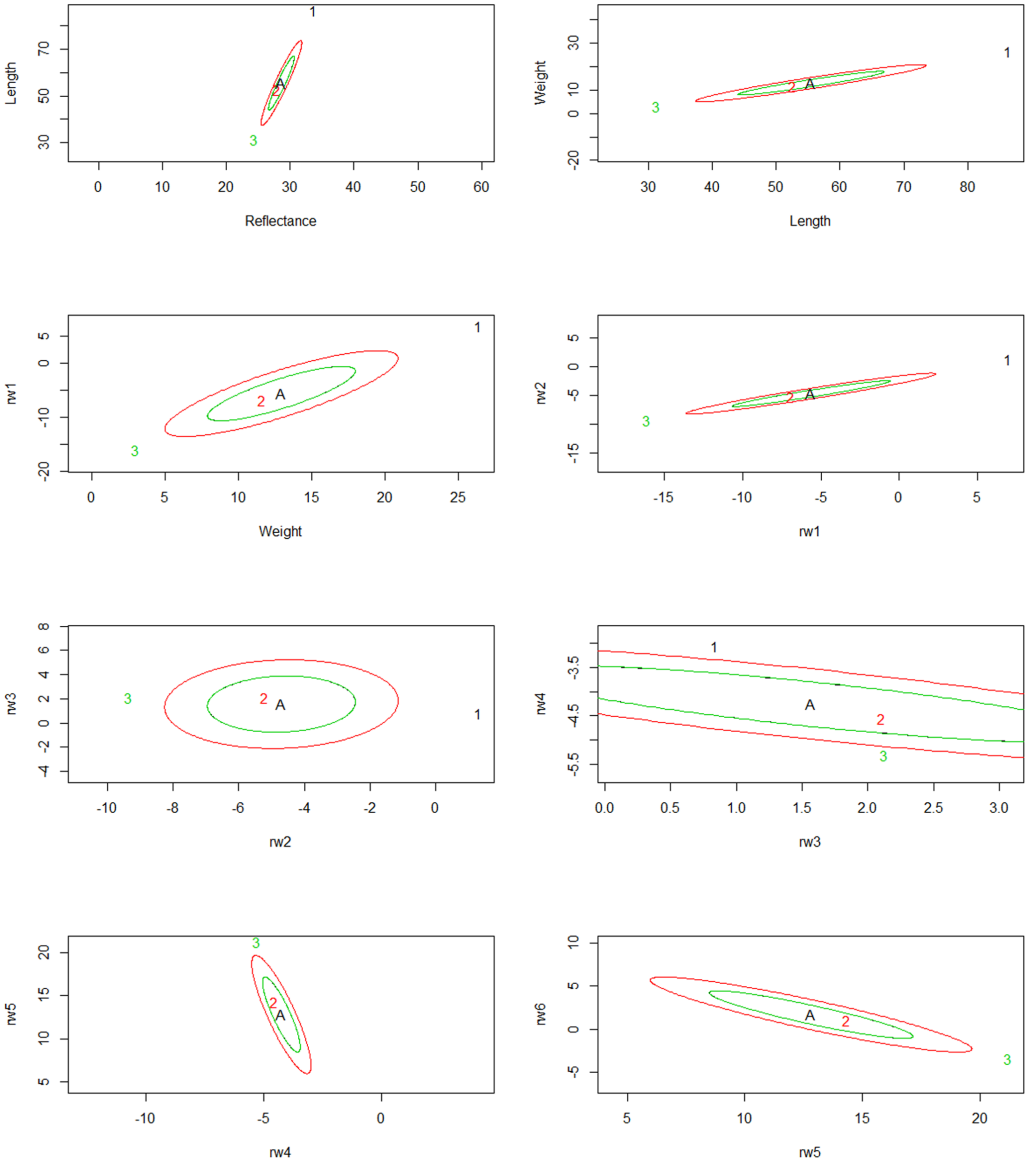
Phenotypic trait distributions for the mean ancestral (A) genotype for trait pairs under neutral expectations at sampling period 1, together with observed population trait values in Assinica (1), Iron River (2), and Siskiwit (3).

**Figure 4:**
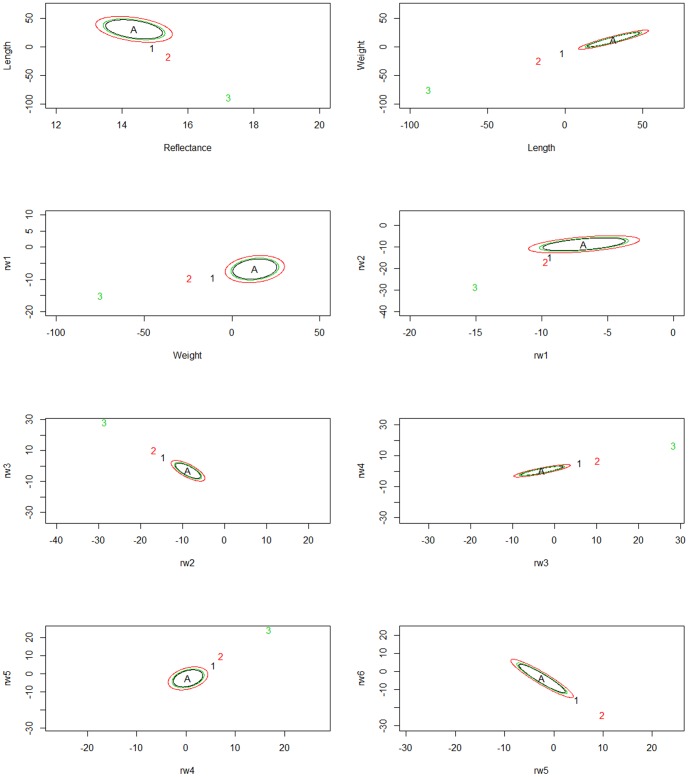
Phenotypic trait distributions for the mean ancestral (A) genotype for trait pairs under neutral expectations at sampling period 2, together with observed population trait values in Assinica (1), Iron River (2), and Siskiwit (3).

**Figure 5:**
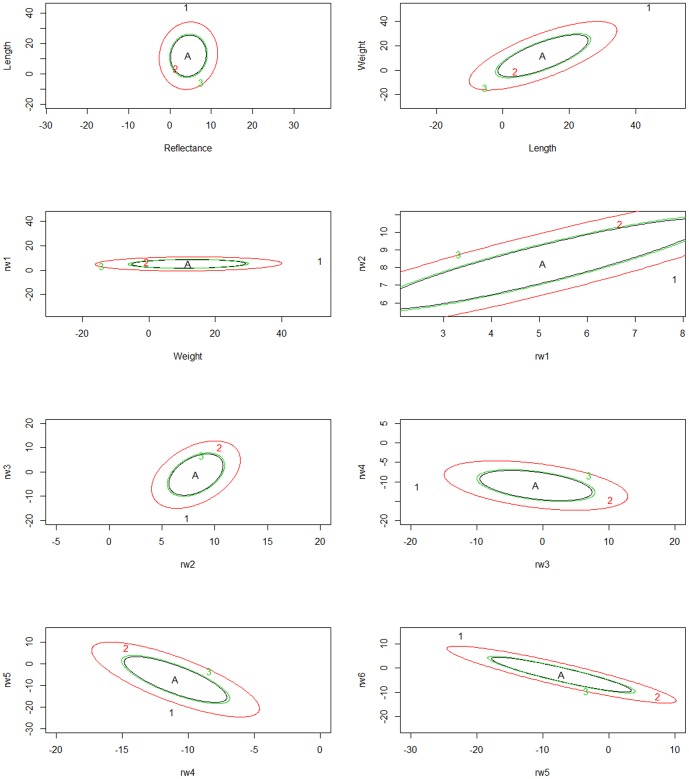
Phenotypic trait distributions for the mean ancestral (A) genotype for trait pairs under neutral expectations at sampling period 3, together with observed population trait values in Assinica (1), Iron River (2), and Siskiwit (3).

In the multivariate analysis of all traits at each time point, population divergence was attributed to divergent selection (Time 1 S = 1, Time 2 S = 1, Time 3 S = 1). To visualize population divergence based on multivariate traits, 95% credible intervals around the ancestral mean genotypes under random genetic drift are drawn for pairs of traits. The observed mean phenotype is plotted as a point to visualize where each population falls with respect to expectations under drift, and with respect to current populations (see Fig. 3). Each population has a corresponding ellipse (identified as being the same color) that represents the 95% posterior density for the additive genetic means expected as a result of genetic drift from the ancestral mean. Populations with trait mean values outside their ellipses show divergent selection while populations whose traits are contained inside the ellipses exhibit differentiation consistent with drift. At the first sampling period, there is evidence for divergent selection in Assinica (population 1) for all traits, and for Iron River (population 3) for all traits except for a single pair of measurements for morphology (relative warps 5 and 6) (Figure 3). At the second sampling period, all populations showed signatures of selection compared to neutral expectations from ancestral mean genotypes (Figure 4). Finally, at the third sampling period when the fish were just over one year old, additive genetic trait distributions generally show a pattern where Iron River and Assinica have diverged from the ancestral mean genotype in a manner consistent with selection, while Siskiwit trait distributions fall within the expectations of divergence due to drift (Figure 5). The Assinica and Iron River populations were consistently in opposing ends of the ellipses at sampling periods 1 and 3 (Figures 3 and 5), indicating selection in different directions. Moreover, this trend shows selection for larger size and greater reflectance (i.e. more silver in body coloration) in Assinica, and for smaller size and darker coloration in Iron River. At time 2, visualization of ellipses for traits showed that selection was acting on all three populations. In this case, all populations showed selection for trait pairs in the same relative directions but differed in magnitude in trait evolution from the ancestral mean genotype; Iron River showed a greater divergence from neutral expectations than the Assinica or the Siskiwit populations (Figure 4). Because the effects of single traits cannot be elucidated in the multivariate driftsel analysis, we also ran driftsel for each trait at each time point individually, with different population groupings. In contrast to the multivariate analyses, results for the majority of traits in the post hoc univariate tests were consistent with differentiation due to genetic drift (Table 1), perhaps illustrating the lack of power in detecting selection when considering only a single trait. When all three populations were compared, divergent selection was found for length and weight at all time points while other traits showed divergence consistent with drift. Pairwise population comparisons were largely consistent with these results, and suggest that the difference between Assinica and Iron River are largely driving the results observed when considering all populations and traits together. Raw data used for the driftsel analyses are provided in Tables S3, S4, and S5 for each of the three time points).

**Table 1:** Signals of selection (S from driftsel) for univariate traits at each time point for different groupings of populations.

Population Comparison	Trait	Time 1	Time 2	Time 3
All Populations	Length	**1.00**	**1.00**	**1.00**
	Weight	**0.90**	**1.00**	**1.00**
	Reflectance	0.69	0.79	0.81
	RW1	0.61	0.58	0.49
	RW2	0.42	0.29	0.43
	RW3	0.59	0.52	0.60
	RW4	0.43	0.55	0.74
	RW5	0.52	0.54	0.62
	RW6	0.28	0.31	0.39
Assinica Siskiwit	Length	**0.93**	**0.99**	**0.99**
	Weight	0.71	**0.98**	**1.00**
	Reflectance	0.68	0.73	0.78
	RW1	0.52	0.43	0.50
	RW2	0.47	0.40	0.44
	RW3	0.67	0.62	0.62
	RW4	0.39	0.43	0.41
	RW5	0.46	0.52	0.61
	RW6	0.42	0.40	0.39
Siskiwit - Iron River	Length	0.69	0.79	0.86
	Weight	0.46	0.63	**0.96**
	Reflectance	0.44	0.43	**0.90**
	RW1	0.41	0.45	0.50
	RW2	0.52	0.39	0.50
	RW3	0.41	0.44	0.48
	RW4	0.47	0.63	0.69
	RW5	0.62	0.65	0.65
	RW6	0.41	0.43	0.43
Iron River Assinica	Length	**1.00**	**1.00**	**1.00**
	Weight	**0.94**	**1.00**	**1.00**
	Reflectance	0.74	0.81	0.43
	RW1	0.68	0.65	0.43
	RW2	0.39	0.39	0.44
	RW3	0.59	0.50	0.54
	RW4	0.48	0.56	0.73
	RW5	0.47	0.43	0.41
	RW6	0.39	0.43	0.46

RW  =  relative warp from analysis of morphology.

## Discussion

In this study quantitative genetic variation was examined in morphological traits within and among *S. fontinalis* strains that have been used currently or historically for stocking in conservation and management efforts in the Lake Superior Basin. The goal of this study was to determine the pattern of divergence in morphological characters over time, and whether there were differences in the patterns or trends for selection on these multivariate quantitative traits for size, shape, and body coloration over the course of development.

The present study builds on a prior study examining heritability of morphological traits relative to the ecology and history of the putative source populations from which the hatchery strains were derived [23]. Though the hatchery strains examined herein do not provide independent replicates of migratory and non-migratory life histories of brook trout, we were still interested in whether or not there were differences in these hatchery populations in body coloration that may have been historically adaptive in the environments from which they were derived (i.e. stream resident vs. migratory between streams and lakes). Though the developmental timing of migration in brook trout that migrate from natal streams to lakes has not been well studied, the three developmental time points measured in this study were meant to capture the possible timing of development in which a migratory salmonid changes from a stream-dwelling parr to a migratory smolt. During this transition, anadromous individuals lose their dark parr marks and their body becomes more silver in coloration [40]. Although the populations used in this study were not anadromous, the progenitors of the Siskiwit and Assinica strains were lake migratory populations while the Iron River strain was founded from a stream resident population. There have been demonstrated similarities in morphology and coloration of fish populations migrating within freshwater habitats when comparing to anadromous populations [29], [30] and lake migratory brook trout generally migrate out of streams in the fall of their first or second year similar to anadromous salmonids [41], [42]. There was a general increase in reflectance developmentally (though note that actual values from the third time point are not comparable to the first two), and Assinica consistently had much higher reflectance than the other two strains in the study, while the Iron River strain, derived from a stream resident population, had consistently darker coloration than the other two strains. This divergence in coloration is consistent with diversifying selection, though was not apparent in univariate analyses. The hatchery strains examined herein have been maintained in hatcheries with variable strategies for maintenance of genetic variation, and for variable lengths of time. It is unknown if the selection in multivariate traits is due to divergence artificial selection in the hatchery environments, vs. natural selection in the source populations without further exploration of the same traits in the natural systems from which they were derived.

Historically, tests for quantitative trait divergence between populations were largely limited to the analyses of single traits, and many populations were required to gain sufficient power needed to reject the null model of divergence due to neutral processes (see [2], [3], [38] for review). In this study, the multivariate analyses examining quantitative trait divergence in three hatchery strains shows marked differentiation in multivariate traits for body size, shape, and coloration could be attributed to divergent selection. Results from the multivariate analysis suggest that all traits were undergoing selection at all time points, however the populations exhibiting selection as well as the directions of selection varied across development. At both time points 1 and 3, the Assinica and Iron River strains had additive genetic means for trait pairs outside of those expected under neutral expectations; however, the direction of selection from the putative ancestral populations were in opposite directions from the ancestral mean genotype, generally indicating selection in the two populations in opposing directions relative to the ancestral mean. For example, the observed additive genetic means for length, weight, and reflectance were larger at all time points for Assinica when compared to the other two populations, while Iron River was consistently smaller. Only at the second time point did the Siskiwit strain show quantitative trait means consistent with the actions of divergent selection, and this could be due to a true difference developmentally in selection for traits ontogenetically, or due to relatively lower power (smaller numbers of families and sample sizes within families) than for the other two strains. The second time point shows a contrasting pattern to the first and third time point, in that all populations exhibit quantitative trait means consistent with divergent selection, but all have diverged in the same direction with respect to the theoretical ancestral mean genotype, just to a different degree. The Assinica again was the most divergent, and has also been in the hatchery environment, without integration with gametes from the natural population since 1962. Without additional populations represented from throughout the range, and without contrasting patterns between natural source and hatchery populations, it cannot be known whether this observation is due to extreme artificial selection in Assinica relative to the Iron River and Siskiwit strains, which have markedly different histories in the hatchery.

In univariate analyses and pairwise population comparisons however, it appeared that the multivariate results were largely driven by divergent selection for length and weight. Differences between length and weight were attributed to divergent selection at most time points for most population comparisons; non-significant results for these traits were concentrated in the Siskiwit-Iron River comparison. The pattern of results indicates that by the final time point, all populations have diverged in length and weight due to selection but at earlier time points, divergent selection is occurring between the Assinica population and each of the other populations but not between the Siskiwit and Iron River populations. The divergence at early life stages between the Assinica and other populations may be due to divergent selection on their progenitor strains but may also be an artifact of the differing lengths of time that the populations have spent in hatcheries. The Assinica strain has been maintained in hatcheries much longer than either the Siskiwit or Iron River strains, and increased growth rates are often directly or indirectly selected for in hatchery environments [43]. The Siskiwit and Iron River strains may not have been exposed to as much domestication selection due to their more recent hatchery introduction, as well as changes in hatchery management practices that have attempted to minimize inadvertent domestication selection. Without testing individuals from wild populations it is impossible to attribute divergence to natural or artificial selection.

The univariate analysis of single traits in this framework showed an overwhelming signature of divergence in quantitative traits that was consistent only with neutral expectations. There are several possible explanations for this. First, our study may simply not have had the power to detect divergent selection. Although driftsel yielded results showing divergent selection, simulation tests in [5] showed that increasing the number of populations would have increased the power to detect divergent selection. In addition, separating traits from the multivariate analysis into univariate analyses results in loss of power and can lead to failure to detect signatures of selection [6]. Second, because the populations used in this study have all been maintained in hatchery environments for varying degrees of time any differences that initially existed between the populations may have been minimized. Assuming hatchery environments are similar, this could lead to the same results where divergence in many traits is consistent with drift. Third, differences between populations for the traits in question may have been due to drift rather than selection. Lake migratory *S. fontinalis* undertake migrations that are typically much shorter in distance and duration than other anadromous salmonid migrations and tend to stay near shore in shallow waters [44], [45]. While the specific differences between the near-shore habitat and riverine habitat are unknown, the dichotomy between these habitats is reduced compared to the ocean and river habitats that other salmonids inhabit. This could result in reduced selection for adaptive divergence between stream resident and lake migratory *S. fontinalis* relative to other resident and ocean migratory salmonids. Finally, while heritable, each individual relative warp explained a small amount of variance in overall body shape. To see if measures based on gross morphological differences might give different results, variation in body depth, caudal peduncle height, and caudal peduncle length between populations were also analyzed in driftsel. In each case divergence was attributed to drift rather than selection (results not shown).

## Conclusions

Currently, few Q_ST_ F_ST_ analyses, or more generally studies on quantitative trait divergence, have examined traits across multiple developmental stages in an organism. Quantitative trait divergence, the result of which is consistent with selection, was evident in the first year of life, and persisted in the three developmental time points examined here. The power of the multivariate approach for examination of quantitative trait divergence is much improved over univariate analyses and traditional Q_ST_ F_ST_ analyses. Multivariate analyses suggest that most all traits in the Assinica and Iron River populations have diverged due to selection, but univariate analyses suggest that divergence due to selection is the most evident for length and weight. Selected differences could be due to domestication effects in hatcheries but without testing the progenitor populations; however it is impossible to determine if this divergence is reflective of wild populations and natural selection in the wild or arose over time by artificial selection in hatcheries.

## Supporting Information

Table S1 **Primer and PCR details for** ***S. fontinalis*** **microsatellites used in this study.**
(DOCX)

Table S2 **Raw genotype data.**
(XLSX)

Table S3 **Phenotypic raw data for time period 1.**
(XLSX)

Table S4 **Phenotypic raw data for time period 2.**
(XLSX)

Table S5 **Phenotypic raw data for time period 3.**
(XLSX)

## References

[1] Merila J, Crnokrak P (2001) Comparison of genetic differentiation at marker loci and quantitative traits. Science 14:892–903.

[2] Leinonen T, O'Hara RB, Cano JM, Merilä J (2008) Comparative studies of quantitative trait and neutral marker divergence: a meta-analysis. J Evol Biol 21:1–17 10.1111/j.1420-9101.2007.01445.x 18028355

[3] Whitlock MC (2008) Evolutionary inference from Q_ST_ . Mol Ecol 17:1885–1896 10.1111/j.1365-294X.2008.03712.x 18363667

[4] O'Hara RB, Merila J (2005) Bias and Precision in Q_ST_ estimates: Problems and some solutions. Genetics 171:1331–1339 10.1534/genetics.105.044545 16085700 PMC1456852

[5] Ovaskainen O, Karhunen M, Zheng C, Arias JMC, Merilä J (2011) A new method to uncover signatures of divergent and stabilizing selection in quantitative traits. Genetics 189:621–632 10.1534/genetics.111.129387 21840853 PMC3189809

[6] Karhunen M, Merilä J, Leinonen T, Cano JM, Ovaskainen O (2013) driftsel: an R package for detecting signals of natural selection in quantitative traits. Mol Ecol Resour 13:746–754 10.1111/1755-0998.12111 23656704

[7] Wright S (1931) Evolution in Mendelian populations. Genetics 16:97–158. 17246615 10.1093/genetics/16.2.97 PMC1201091

[8] Lande R (1976) Natural selection and random genetic drift in phenotypic evolution. Evolution 30:314–334. 28563044 10.1111/j.1558-5646.1976.tb00911.x

[9] D'Amelio S, Wilson C (2008) Genetic population structure among source populations for coaster brook trout in Nipigon Bay, Lake Superior. Trans Am Fish Soc 137:1213–1228 10.1577/T05-275.1

[10] Mavarez J, Audet C, Bernatchez L (2009) Major disruption of gene expression in hybrids between young sympatric anadromous and resident populations of brook charr (*Salvelinus fontinalis* Mitchill). J Evol Biol 22:1708–1720 10.1111/j.1420-9101.2009.01785.x 19549137

[11] Wilson CC, Stott W, Miller L, D'Amelio S, Jennings MJ, et al (2008) Conservation genetics of Lake Superior brook trout: issues, questions, and directions. North Am J Fish Manag 28:1307–1320 10.1577/M05-190.1

[12] Huckins CJ, Baker EA, Fausch KD, Leonard JBK (2008) Ecology and life history of coaster brook trout and potential bottlenecks in their rehabilitation. North Am J Fish Manag 28:1321–1342 10.1577/M05-191.1

[13] Cooper AM, Miller LM, Kapuscinski AR (2009) Conservation of population structure and genetic diversity under captive breeding of remnant coaster brook trout (*Salvelinus fontinalis*) populations. Conserv Genet 11:1087–1093 10.1007/s10592-009-9841-0

[14] Leonard JBK, Stott W, Loope DM, Kusnierz PC, Sreenivasan A (2013) Biological consequences of the coaster brook trout restoration stocking program in Lake Superior tributaries within Pictured Rocks National Lakeshore. North Am J Fish Manag 33:359–372 10.1080/02755947.2012.754801

[15] Kostow KE, Marshall AR, Phelps SR (2003) Naturally spawning hatchery steelhead contribute to smolt production but experience low reproductive success. Trans Am Fish Soc 132:780–790 10.1577/T02-051

[16] Leider SA, Hulett PL, Loch JJ, Chilcote MW (1990) Electrophoretic comparison of the reproductive success of naturally spawning transplanted and wild steelhead trout through the returning adult stage. Aquaculture 88:239–252 10.1016/0044-8486(90)90151-C

[17] Taylor EB (1991) A review of local adaptation in Salmonidae, with particular reference to Pacific and Atlantic salmon. Aquaculture 98:185–207.

[18] Jensen LF, Hansen MM, Pertoldi C, Holdensgaard G, Mensberg K-LD, et al (2008) Local adaptation in brown trout early life-history traits: implications for climate change adaptability. Proc R Soc B Biol Sci 275:2859–2868 10.1098/rspb.2008.0870 PMC2605839 18755673

[19] Carlson SM, Seamons TR (2008) A review of quantitative genetic components of fitness in salmonids: implications for adaptation to future change. Evol Appl 1:222–238 10.1111/j.1752-4571.2008.00025.x 25567628 PMC3352437

[20] Fraser DJ, Bernatchez L (2005) Adaptive migratory divergence among sympatric brook charr populations. Evolution 59:611–624. 15856703 10.1554/04-346

[21] Seamons TR, Hauser L, Naish KA, Quinn TP (2012) Can interbreeding of wild and artificially propagated animals be prevented by using broodstock selected for a divergent life history? Evol Appl 5:705–719 10.1111/j.1752-4571.2012.00247.x 23144657 PMC3492896

[22] Præbel K, Knudsen R, Siwertsson A, Karhunen M, Kahilainen KK, et al (2013) Ecological speciation in postglacial European whitefish: rapid adaptive radiations into the littoral, pelagic, and profundal lake habitats. Ecol Evol 3:4970–4986 10.1002/ece3.867 24455129 PMC3892361

[23] Varian A, Nichols KM (2010) Heritability of morphology in brook trout with variable life histories. PLoS One 5:e12950 10.1371/journal.pone.0012950 20886080 PMC2944874

[24] Newman LE, DuBois RB, Halpern TN (2003) A brook trout rehabilitation plan for Lake Superior. Ann Arbor, MI: Great Lakes Fishery Commission Miscellaneous Publication 2003-03.

[25] Flick W (1977) Some observations, age, growth, food-habits and vulnerability of large brook trout (*Salvelinus fontinalis*) from four Canadian lakes. Nat Can 104:353–359.

[26] Van Offelen H, Krueger C, Schofield C (1993) Survival, growth, movement, and distribution of two brook trout strains stocked into small Adirondack streams. North Am J Fish Manag 13:86–95.

[27] Sutton TM, Pangle KL, Greil RW (2002) Hatchery performance attributes of Nipigon, Assinica, and Iron River strains of age-0 brook trout. N Am J Aquac 64:188–194.

[28] Angers B (1995) Specific microsatellite loci for brook charr reveal strong population subdivision on a microgeographic scale. J Fish Biol 44:388–185 10.1111/j.1095-8649.1995.tb06054.x

[29] Boula D, Castric V, Bernatchez L (2002) Physiological, endocrine, and genetic bases of anadromy in the brook charr, *Salvelinus fontinalis*, of the Laval River (Quebec, Canada). Environ Biol Fishes 64:229–242.

[30] Bourke P, Magnan P, Rodriguez MA (1997) Individual variations in habitat use and morphology in brook charr. J Fish Biol 51:783–794.

[31] Nichols KM, Edo AF, Wheeler PA, Thorgaard GH (2008) The genetic basis of smoltification-related traits in *Oncorhynchus mykiss* . Genetics 179:1559–1575 10.1534/genetics.107.084251 18562654 PMC2475755

[32] Rohlf FJ (2007) tpsRelw, relative warps analysis, version 1.45 Department of Ecology and Evolution, State University of New York at Stony Brook.

[33] Goudet J (1995) Fstat (Version 1.2): a computer program to calculate F-statistics. J Hered 86:485–486 10.1093/jhered/91.4.348

[34] Cockerham CC (1969) Variance of Gene Frequencies. Evolution 23:72–84. 28562963 10.1111/j.1558-5646.1969.tb03496.x

[35] Cockerham CC (1973) Analyses of Gene Frequencies. Genetics 74:679–700. 17248636 10.1093/genetics/74.4.679 PMC1212983

[36] Karhunen M (2013) RAFM Reference Manual.

[37] Plummer M, Best N, Cowles K, Vines K, Sarkar D, et al**.** (2012) CODA Reference Manual.

[38] Whitlock MC, Guillaume F (2009) Testing for spatially divergent selection: comparing Q_ST_ to F_ST_ . Genetics 183:1055–1063 10.1534/genetics.108.099812 19687138 PMC2778959

[39] D'Amelio S, Mucha J, Mackereth R, Wilson CC (2008) Tracking coaster brook trout to their sources: combining telemetry and genetic profiles to determine source populations. North Am J Fish Manag 28:1343–1349 10.1577/M05-193.1

[40] Hoar WS (1976) Smolt transformation: Evolution, behavior, and physiology. J Fish Res Board Canada 33:1233–1252.

[41] Huckins CJ, Baker EA (2008) Migrations and biological characteristics of adfluvial coaster brook trout in a south shore Lake Superior tributary. Trans Am Fish Soc 137:1229–1243 10.1577/T05274.1

[42] Theriault V, Dodson JJ (2003) Body size and the adoption of a migratory tactic in brook charr. J Fish Biol 63:1144–1159 10.1046/j.1095-8649.2003.00233.x

[43] Hutchings JA, Fraser DJ (2008) The nature of fisheries- and farming-induced evolution. Mol Ecol 17:294–313 10.1111/j.1365-294X.2007.03485.x 17784924

[44] Mucha JM, Mackereth RW (2008) Habitat use and movement patterns of brook trout in Nipigon Bay, Lake Superior. Trans Am Fish Soc 137:1203–1212 10.1577/T05-273.1

[45] Mucha JM (2003) Habitat use, movement patterns, and home ranges of coaster brook trout in Nipigon Bay, Lake Superior. M. Sc. Thesis, Lakehead University.

