# Correction: Genetic and Morphological Divergence in Three Strains of Brook Trout *Salvelinus fontinalis* Commonly Stocked in Lake Superior

**DOI:** 10.1371/journal.pone.0118278

**Published:** 2015-03-18

**Authors:** The PLOS ONE Staff

There are errors in Fig. 2. The asterisks denoting statistical significance are missing from the graph. The authors have provided a corrected version here.

**Fig 2:**
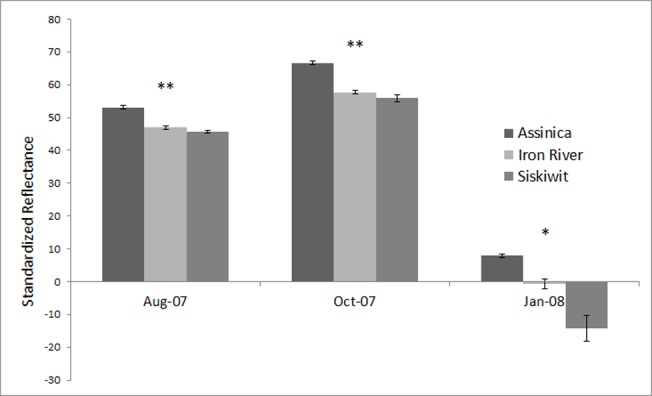
Standardized reflectance for each strain across sampling time points. *Indicates that all three strains are significantly different. **Indicates that Assinica is significantly different from both Siskiwit and Iron River. Magnitude of reflectance for the last time point is not directly comparable to the first two as a different intensity standard was used.
