# Supplementary material for: Genetic and Morphological Divergence in Three Strains of Brook Trout Salvelinus fontinalis Commonly Stocked in Lake Superior
Source: PLoS One. 2014 Dec 5;9(12):e113809. doi: 10.1371/journal.pone.0113809 (PMC4257586; doi:10.1371/journal.pone.0113809)
Supplement: Table S1 — Primer and PCR details for S. fontinalis microsatellites used in this study. (DOCX) [file pone.0113809.s001.docx]

Table S1. Primer and PCR details for *S. fontinalis* microsatellites used in this study.

| Locus | Primer Concentration **(μM)** | 5’ Modification | Annealing Temperature (°C) | GenBank Accession Number |
| --- | --- | --- | --- | --- |
| *Sfo*8 | 0.20 | M13 | 57 | U50305 |
| *Sfo*12 | 0.20 | M13 | 57 | U50302 |
| *Sfo*18 | 0.20 | M13 | 57 | U50303 |
| *Sfo*C24 | 0.20 | M13 | 57 | AY168187 |
| ***Sfo*C28** | 0.20 | M13 | 57 | AY168188 |
| ***Sfo*C79** | 0.06 | VIC | 57 | AY168190 |
| ***Sfo*C86** | 0.06 | NED | 62 | AY168191 |
| ***Sfo*C88** | 0.06 | PET | 59 | AY168192 |
| ***Sfo*C113** | 0.20 | M13 | 57 | AY168193 |
| ***Sfo*C115** | 0.20 | VIC | 57 | AY168194 |
| ***Sfo*C129** | 0.16 | FAM | 57 | AY168195 |
| ***Sfo*D75** | 0.16 | PET | 57 | AY168197 |
| ***Sfo*D91** | 0.20 | VIC | 54 | AY168198 |
| ***Sfo*D100** | 0.16 | NED | 60 | AY168199 |
